# Working conditions and mental illness among nursing workers

**DOI:** 10.47626/1679-4435-2022-980

**Published:** 2024-08-05

**Authors:** Mirian Caroline Pereira, Leonardo Dresch Eberhardt, Manoela de Carvalho

**Affiliations:** 1 Saúde Pública em Região de Fronteira, Universidade Estadual do Oeste do Paraná, Foz do Iguaçu, PR, Brazil; 2 Faculdade de Enfermagem, Universidade Estadual de Campinas (UNICAMP), Campinas, SP, Brazil; 3 Departamento de Saúde Pública, Faculdade de Medicina, Universidade Estadual Paulista “Júlio de Mesquita Filho” (UNESP), Botucatu, SP, Brazil

**Keywords:** occupational health, mental health, nursing staff, saúde do trabalhador, saúde mental, recursos humanos de enfermagem

## Abstract

The growth of mental illness has aroused the interest of the occupational health area in the study of the relationship between work and mental health. Among health workers, nursing represents the largest contingent of workforce in the sector and, due to frequent exposure to numerous stressors, they present a significant increase in work-related mental illness. The objective of this study was to identify the most frequent illness and mental distress processes among nursing professionals in Brazil and relate them to working conditions and coping strategies described in recent scientific literature. The integrative review was conducted in electronic health databases, in May 2020, and resulted in the selection and analysis of 17 studies. The results show that the hospital environment and its working conditions, overload of activities, precarious working conditions, short deadlines to carry out activities and conflicting relationship with the team and users constitute the main scenario of studies on illness and mental distress of nursing workers. The most frequent illness and mental distress processes involve the consequences of stress such as: anxiety, demotivation, bad mood, body aches, musculoskeletal disorders, irritability, alteration of menstrual flow, insomnia, attention and concentration deficit, gastric and duodenal ulcers, fatigue, migraines, among others. The strategies adopted by workers to minimize work stress are predominantly individual, pointing to a gap in studies, or in reality itself, about collective strategies.

## Abstract (pt)

O crescimento do adoecimento psíquico tem despertado o interesse da área de Saúde do Trabalhador no estudo da relação entre trabalho e saúde mental. Entre os profissionais da saúde, a enfermagem representa o maior contingente da força de trabalho no setor, e, devido à frequente exposição a inúmeros fatores estressores, esses trabalhadores apresentam um aumento significativo do adoecimento mental relacionado ao trabalho. O objetivo deste estudo foi identificar os processos de adoecimento e sofrimento mental mais frequentes entre profissionais da enfermagem no Brasil e relacioná-los às condições de trabalho e às estratégias de enfrentamento descritas na literatura científica recente. A revisão integrativa foi conduzida em bases de dados eletrônicas na área da saúde, em maio de 2020, e resultou na seleção e análise de 17 estudos. Os resultados mostram que o ambiente hospitalar, a sobrecarga de atividades, as condições de trabalho precárias, os prazos curtos para realizar as atividades e a relação conflituosa com a equipe e os usuários constituem o principal cenário dos estudos sobre o adoecimento e sofrimento mental dos trabalhadores de enfermagem. Os processos de adoecimento e sofrimento mental mais frequentes envolvem as consequências do estresse, como ansiedade, desmotivação, mau humor, dores no corpo, distúrbios osteomusculares, irritabilidade, alteração do fluxo menstrual, insônia, déficit de atenção e concentração, úlceras gástricas e duodenais, fadiga, enxaquecas, entre outros. As estratégias adotadas pelos trabalhadores para minimizar o estresse no trabalho são predominantemente individuais, apontando para uma lacuna de estudos ou da própria realidade acerca de estratégias coletivas.

## INTRODUCTION

Historically, human relationships with work in a capitalist society can lead to physical, psychological, and emotional distress and illness.^1^ In recent decades, mental health problems have grown among the working population in Brazil, with little recognition by public bodies and health care providers.^2-4^

The rise of mental illness has sparked interest in the field of occupational health in terms of studying the relationship between work and mental health. Seligmann-Silva et al.^5^ identify three schools of thought in the literature about this relationship: the first has its roots in the theory of stress at work^6-9^; the second is based on psychoanalytic references^10,11^; and the third is grounded in the theory of historical-dialectical materialism and is nucleated by the concept of “wear and tear”.^12,13^ Subsequently, in addition to these approaches,^5^ a fourth approach emerged, which is the clinical activity of Yves Clot.^14^

The stress-at-work approach is also known as the demand-control model.^9^ According to this model, based on Karasek’s studies,^6^ stress at work is related to work settings with high demand and low control (low decision-making latitude) among workers. Studies based on this model have found a relationship between stress at work and physical and mental health problems, such as mental disorders and cardiovascular and cerebrovascular problems, among others.^6-9,15,16^

The second view, based on the studies of Christophe Dejours^10,11^ on the psychopathology and psychodynamics of work, considers stress as an attempt to adapt, which may or may not evolve into pathological issues, such as the most significant anxiety and depression disorders, for example panic disorder, obsessive-compulsive disorder and depression.^17^ Although stress is not only related to work, its relevance in everyday life makes it one of the main triggers of stress.^18^

In this context, the term “pathology” is commonly used to define a psychopathological decompensation, that is, a rupture in mental stability which is expressed in the onset of a mental illness.^17^ The pathological factor at work arises when distress is no longer bearable, i.e. when the worker has used all their intellectual and psychoaffective resources to comply with the activity and the demands imposed by the organization, but finds themselves powerless to adapt and/or transform their work.^19^

The theory based on Laurell & Noriega’s wearand-tear-reproduction^12^ considers wear and tear as the “loss of effective and/or potential biological and psychological capacity,”^12^ caused by workloads and the processes of social reproduction of the workforce. In this context, workers develop forms of defense and resistance to cope with harmful working conditions.^13^

Finally, based on Yves Clot^14^ studies, the last school of thought on the relationship between work and mental health is centered on the concept of “real activity” and develops tools such as self-confrontation and coaching. It focuses on increasing workers’ power to act.^14,20^

The novel coronavirus pandemic that spread between the end of 2019 and the beginning of 2020 has caused a series of social and economic changes, such as recession, isolation, rising unemployment, and exposure to the virus, triggering fear, mourning, insecurity, and a loss of perspectives. In this sense, in addition to respiratory conditions such as severe acute respiratory syndrome, the COVID-19 pandemic may be related to the emergence and worsening of a wide range of human health problems, including mental health problems.^21,22^

During the pandemic, different categories of workers continued to work in essential activities. Even with the safety protocols adopted, workers were exposed to infection, illness and, potentially, death from COVID-19.^23^

Among health workers, nurses represent the largest contingent of the workforce, and despite being defined as the science of comprehensive and integrative health care, these workers, due to frequent exposure to numerous stressors, show a significant increase in work-related mental illness.^24^ Health care personnel, especially nurses, have been at the forefront of the fight against the pandemic, with high rates of infection and death due to the novel coronavirus, with possible repercussions on mental health.^25^ Deaths among nurses in Brazil were one third of all deaths worldwide.^26^

In light of the importance and relevance of the issue of mental illness among nurses, a bibliographical search was conducted to answer this question: What are the working conditions related to mental health problems among nurses and the coping strategies described in the scientific literature? The study aimed to identify the most frequent processes of illness and mental distress among nurses in Brazil and link them to the working conditions and coping strategies described in recent scientific literature. Determining current knowledge on the subject can contribute to the development of research and interventions aimed at prevention and health promotion.

## METHODS

This study is an integrative literature review that included six stages: (1) defining the question for the review (guiding question); (2) searching for and screening the studies that comprise the study sample; (3) surveying the characteristics sought in the reviewed studies; (4) analyzing the findings according to empirically constructed categories; (5) interpreting and discussing the results; and, finally, (6) disseminating them.^27,28^

The data was collected from the Virtual Health Library (VHL) website in May 2020, using the following filters available in the database: “full text available,” “Portuguese language interface,” and “publication interval of 5 years.” As this study deals with occupational health, the selection criteria were studies with methods that effectively included nurses in their samples, ensuring the expression of workers as protagonists to understand their healthdisease process.

The keywords used were based on the Medical Subject Headings (MeSH) to ensure control of the structured vocabulary and the identification of words associated with the study. PICO, a method used to intervene and extract the fundamental elements of the review, represented by Population, Intervention, Comparison, and Outcomes, combined with the Boolean AND function, was used as follows: “trabalhador da enfermagem” (P), “condições de trabalho” (I), “sofrimento psíquico” (O), and the general population was used for comparison (C). The review sample excluded theses, dissertations, monographs, review articles, duplicate studies, studies older than five years, studies in a foreign language, or studies with no full text available online.

The search strategy used found 2,078 results. After reading the titles and using the inclusion and exclusion criteria, 2,031 studies were removed, of which 69 were duplicates, 1,253 were theses, dissertations, monographs, and review articles, and 709 did not meet the guiding question. We then read the abstracts of the 47 studies that fit the proposed theme, excluding 19 papers which did not consider the involvement of workers in the study and 11 studies which failed to answer the proposed objectives in the results of the study. The screening process yielded 17 studies that met the objective of the review. They were read in full and included in the final sample of the bibliographical research.

After reading and filing the articles included in the final sample, data analysis and organization included building empirical categories and comparing them with the relevant literature. The results are presented descriptively, grouped into empirical categories.

## RESULTS

Chart 1 shows the general description of the 17 studies selected for the literature search. Of these, 3 were published in 2020, 2 in 2019, 6 in 2018, 1 in 2017, 2 in 2016, and 3 in 2015. As for the methods used in the studies, although different methods were used, 11 qualitative studies were included.

**Chart 1:** Characteristics of the studies included according to title, year of publication, and method (2015-2020)

Study	Title	Year of publication	Study design
A129	Occupational stress among nursing professionals	2018	Descriptive, qualitative, and correlational
A230	Stress and coping strategies among nursing professionals in a family medicine unit	2017	Descriptive, qualitative
A331	Sociodemographic and occupational profile and assessment of mental health conditions of Family Medicine Strategy workers in a municipality in Rio Grande do Sul, RS, Brazil	2016	Cross-sectional, epidemiological, and quantitative
A432	The body speaks: physical and psychological aspects of stress among nursing professionals	2016	Descriptive, qualitative
A533	A negative self-reported health assessment in primary care nursing professionals	2018	Cross-sectional, exploratory
A634	The relevance of self-care for nursing professionals	2015	Descriptive, qualitative
A735	Depression symptoms and drug addiction among nursing professionals	2018	Cross-sectional, quantitative
A836	The influence of job insecurity on the work process and the health of nursing professionals	2018	Qualitative
A937	Armed conflicts and their impact on the health of professionals working in the Family Health Strategy in the city of Rio de Janeiro, RJ, Brazil	2020	Qualitative
A10^38^	Moral distress of nursing professionals in a Psychosocial Care Center	2020	Descriptive, qualitative
A11^39^	Enjoyment and distress in nursing jobs in a pediatric intensive care unit	2019	Descriptive, qualitative
A12^40^	Job satisfaction among nursing professionals in the public health network in a capital city in Brazil	2020	Cross-sectional, quantitative
A13^41^	Impact of burnout syndrome on the quality of life of primary health care nursing professionals	2019	Exploratory, descriptive, qualitative
A14^42^	Stress in nursing professionals: importance of the organizational climate dimension	2018	Qualitative
A15^43^	Association between burnout syndrome, harmful use of alcohol, and smoking among nurses in the ICUs of a university hospital	2018	Qualitative
A16^44^	Does health production lead to illness? The contradictions of working in emergency rooms in public hospitals	2015	Quantitative
A17^45^	Absenteeism among nursing professionals at a university hospital in the state of São Paulo, SP, Brazil	2015	Descriptive, qualitative

Source: Prepared by the authors based on literature search on Virtual Health Library (VHL), May 2020.

Chart 2 shows the working fields and areas of nursing professionals involved in the studies included in the literature review. The working fields or areas the studies covered included primary health care (6), university hospitals (5), intensive care units (3), general hospitals (1), emergency room (1), and psychosocial care centers - CAPS (1). Therefore, the hospital environment was the main setting for studies on mental illness and distress among nursing professionals, with 10 studies published.

**Chart 2:** Working field of nursing professionals in the studies included (2015-2020)

Working field or area	Studies
Primary Health Care	A2^30^; A3^31^; A5^33^; A9^37^; A12^40^; A13^41^
University hospital	A1^29^; A4^32^; A8^36^; A15^43^; A17^45^
Intensive care unit (ICU); pediatric ICU; neonatal ICU	A6^34^; A11^39^; A14^42^
General hospital; emergency public hospital	A735; A1644
Psychosocial care center	A10^38^

The 4 empirical categories constructed from data analysis and compilation are shown in Chart 3, with the corresponding studies.

**Chart 3:** Nursing-related illness and distress, working conditions, and coping strategies according to studies included (2015-2020)

Category		Studies
I	Nursing working conditions and risk factors for stress	A1^29^; A2^30^; A4^32^; A5^33^; A6^34^; A7^35^; A8^36^; A10^38^; A11^39^; A12^40^; A15^43^; A16^44^; A17^45^
II	Consequences of stress on the health of nursing professionals	A1^29^; A2^30^; A3^31^; A4^32^; A5^33^; A6^34^; A7^35^; A8^36^; A9^37^; A10^38^; A12^40^; A13^41^; A14^42^; A15^43^; A16^44^; A17^45^
III	Strategies for coping with illness and distress at work	A2^30^; A6^34^; A7^35^; A10^38^; A11^39^; A12^40^; A14^42^; A15^43^
IV	Use of psychoactive substances among nursing professionals	A7^35^; A12^40^; A15^43^

Category I, “Nursing working conditions and risk factors for stress,” highlighted the following among the factors that trigger stress at work: excessive workload; precarious working conditions; short deadlines to complete tasks; and conflicting relationships with staff and users.

The participants included in the previous studies showed signs and symptoms of physical and mental fatigue, muscle pain, and an imbalance in the weight cycle. These symptoms contribute to professional stress and reduce the quality of life of nursing professionals in their work environment.^29,30,32-38^

These studies also highlight the prevalence of feminization in the nursing profession, which consists mostly of married women with children and working more than one job.

In addition, these studies found that stressful roles in the career, combined with the work shift (morning and evening), and the gap between nursing theory and practice in the workplace, can trigger distress and mental illness.39,40,43,45

Category II, “Consequences of stress on the health of nursing professionals,” shows that health stress among nursing professionals can lead to physical and mental illness.^30-34^ Among the main consequences of stress are sensations and feelings such as tension, fear, anguish, anxiety, demotivation, bad mood, body aches, musculoskeletal disorders, irritability, changes to menses, insomnia, attention and concentration deficits, gastric and duodenal ulcers, fatigue, migraines, gastrointestinal disorders, and low immunity.^35-38,40-45^ In other words, a wide range of health problems related to nursing working conditions can be observed.

Rodrigues & Santos^32^ (A4) point out that:

“Stress has a direct influence on the personal and professional lives of all individuals, and can cause a disruption in the physical balance of the body. This imbalance is directly related to the health and illness of these individuals. The development of a state of stress is intrinsically related to what the individual sees as a stressor. This source of stress can be external, related to the profession, arguments, losses; and internal, such as the state of being, our beliefs and values, the manner of acting.”^32^

In this respect, stress is related to subjective dimensions, referring to the individual, and collective dimensions, related to society, and can have negative repercussions for work performance, and for personal life and society itself.^32^

Lua et al.^33^ (A5) affirm that, among workers directly involved in patient care, the highest rates of emotional exhaustion, depersonalization, and low levels of professional achievement are found among nursing professionals. According to this study, “Feelings of demotivation, discouragement, and emotional fatigue are responses to initial moral distress, and are understood as a consequence of the psychological imbalance these professionals experience when they are faced with barriers to performing actions and behaviors they consider to be appropriate, and are prevented from executing them.”^33^

Category III, “Strategies for coping with illness and distress at work,” includes studies that point to individual and collective strategies nursing professionals use to minimize work-related stress.

Participants in the studies reported the importance of family and professional support networks in promoting mental health care, combined with leisure activities. It is important to maintain interpersonal relationships focused on good family and professional relations. This good relationship becomes an essential component for health promotion.^30,34,35^

According to Oliveira et al.^38^ (A10), leisure helps to relieve the tension resulting from nursing work, as it is a field where workers constantly deal with distress, illness, and death. Constantly living under these circumstances can lead to emotional overload, with feelings of anguish and depressive syndromes emerging, among other mental health problems.

In this literature review, category IV, “Use of psychoactive substances among nursing professionals,” highlighted the use of psychoactive substances as a strategy for coping with working conditions. Junqueira et al.^35^ (A7) made a comparison between men and women in order to survey preferences in the use of psychoactive substances. While men showed a predominance of alcohol and cannabis consumption, related to symptoms suggestive of depression, among women, who make up the majority of the nursing workforce, sedatives were frequently used, associated with feelings of hopelessness, sadness, lack of interest and pleasure, with a potentially increased risk of developing depression.^35^

It is important to consider that the use of tranquilizers in this case refers to the consumption of psychotropic drugs without a medical prescription. This is a very worrying issue in the context of nursing, considering the involvement, ease of access to these drugs, and the high rates of use among professionals.^40^ In addition to tranquillizers, women are more likely to smoke.^43^

## DISCUSSION

According to the results of the bibliographic search, it is clear that nursing professionals’ daily working conditions can lead to physical, psychological, and emotional illness, in addition to a series of poorlycharacterized manifestations, leading to a discussion about “diffuse distress,” which cannot be classified in medical or psychiatric nosology.^46^

A series of elements in the nursing team’s work process can alter the level of stress, such as excessive workloads, a high emotional burden, demands from family members and managers, the vulnerability of patients, the feeling of helplessness in the face of pain and death and the lack of time for leisure and rest, among other significant experiences.^47^

The studies included in this review consider that working conditions in hospitals do not encourage teamwork. Each professional tends to be concerned with performing processes that are only relevant to their professional category, weakening teamwork.^48^ This also undermines the relationships and bonds between team members, which can lead to uncertainty and a lack of essential information to ensure that care runs smoothly^49,50^ and consequently overburdens the nursing team.

In relation to primary care services, nursing professionals reported that Municipal Health Departments are constantly demanding that they meet established goals, making them feel that they have no control over their jobs. This leads to work overload, as it requires a great deal of effort to perform daily duties, plus meeting goals, which can lead to distress and illness.^50^

Work-related overload and altered stress levels, together with workers’ lack of control over their work process, can be interpreted in the light of Karasek’s demand-control model as highly stressful or demanding work, related to a wide range of physical and mental health problems, especially of a chronic nature.^6-9,15,16^

It is additionally demanding for nursing professionals to work shifts, whether in primary health care, hospitals, or CAPS. Working night shifts is considered harmful to workers’ health, as it increases physical and mental exhaustion and musculoskeletal disorders, and hinders family and social life.^48^ This is made all the more significant because nursing has historically been an eminently female profession, with working conditions marked by issues of gender inequality. This is the case with extra working hours, which lead to an accumulation of tasks from different sources. The need to work outside the home together with the need to look after children and family brings about conflicts, leading to both work-related and personal stress in their daily lives. This is an element that becomes a contradictory factor, as family can be both a stress factor and a factor of emotional support and assistance to relieve the tension caused by work.^50,51^

In short, work-related psychosocial factors represent a set of perceptions and experiences. In other words, they consist of interactions between work, the workplace, the conditions of the organization, and the personal characteristics of the worker, their needs, culture, experiences, lifestyle, and their perception of the world.^17^ Among the main psychosocial factors at work that cause stress are aspects of the organization, management, the work process, and human relations.^18^

The pathological factor at work occurs when workers feel powerless to adapt or transform their work, that is, when they have already used all their intellectual and psychoaffective resources to circumvent the distress caused by the demands imposed by their work organization.^19^ It arises in relation to a reduction in the power to act, according to the activity clinic,^12^ or the breakdown of workers’ forms of resistance by management, according to the theory of attrition-reproduction.^14^

Among the defense and resistance strategies, the studies found that nursing professionals try to engage in some kind of leisure activity to deal with tension. These practices act as wellness promoters, enabling positive feelings and sensations to be better prepared to face the uncertainties and tension of the work environment.^50^

However, the individual solutions found by nursing professionals to deal with stress go beyond leisure and family life, involving the use of psychoactive substances, which can lead to changes in work, interpersonal, family, social and health relationships.^52,53^

The use of psychoactive substances by nursing professionals can be considered a strategy of (individual) defense^4^ or mediation of distress,^13^ in which there is an attempt to deal with the problem and its determinants, not confronting them directly. It is noteworthy that strategies of this kind are often the only ones available, but they can cause collateral damage in the long run, including worsening the problem.

Therefore, it is necessary to emphasize the need to overcome (individual) defense strategies in favor of (collective) resistance strategies, in the terms of Seligmann-Silva,^13^ in order to deal with the problems related to the work process and mental health among nursing professionals. Resistance strategies can involve existing organizational forms, such as trade unions, associations and class councils, and the creation of new organizational instruments.

In this review, the study of nursing workers’ (collective) resistance strategies was a gap that may be related to both the scarcity of collective organizing experiences in the category and the lack of scientific interest in this area.

## FINAL CONSIDERATIONS

This literature review has shown that the work processes of nursing professionals are related to physical, psychological, and emotional consequences for their health. The health-disease process of nursing professionals includes the use of strategies to cope with stress and mental distress. These strategies are predominantly individual, involving, on the one hand, family and professional support networks and leisure practices, in order to promote mental health care. On the other hand, the strategies nursing professionals use also include psychoactive substances to deal with work-related stressful daily issues.

This review enabled to synthesize the main forms of illness, working conditions, and the manners nursing professionals cope with them. It also allowed to identify an important gap regarding collective forms of coping in the management of everyday relationships in the workplace.

Another gap refers to the lack of studies incorporating workers who are off work for different reasons. It is therefore suggested that other studies investigate the relationship between sick leave and mental illness in nursing work, mainly because mental health problems lead to prolonged sick leave and are hardly recognized as work-related by public bodies and companies.^5^

Finally, it is important to emphasize the need for empirical studies on the subject in order to deepen the issues raised in this review article. At the moment, an important issue is the mental health of nursing professionals during the COVID-19 pandemic.
